# A “plus one” strategy impacts replication of felid alphaherpesvirus 1, *Mycoplasma* and *Chlamydia,* and the metabolism of coinfected feline cells

**DOI:** 10.1128/msystems.00852-24

**Published:** 2024-09-24

**Authors:** Sara M. Klose, David P. De Souza, Joanne M. Devlin, Rhys Bushell, Glenn F. Browning, Paola K. Vaz

**Affiliations:** 1 Asia-Pacific Centre for Animal Health, Melbourne Veterinary School, University of Melbourne, Melbourne, Victoria, Australia; 2 Metabolomics Australia, Bio21 Institute, University of Melbourne, Melbourne, Victoria, Australia; 3 Department of Veterinary Clinical Sciences, Melbourne Veterinary School, University of Melbourne, Melbourne, Victoria, Australia; University of California Irvine, Irvine, California, USA

**Keywords:** coinfection, metabolomics, herpesvirus, mycoplasma, chlamydia, cats

## Abstract

Coinfections are known to play an important role in disease progression and severity. Coinfections are common in cats, but no coinfection studies have investigated the *in vitro* dynamics between feline viral and bacterial pathogens. In this study, we performed co-culture and invasion assays to investigate the ability of common feline bacterial respiratory pathogens, *Chlamydia felis* and *Mycoplasma felis*, to replicate in and invade into Crandell–Rees feline kidney cells. We subsequently investigated how coinfection of these feline cells with each bacterium (*C. felis* or *M. felis*) and the common feline viral pathogen, felid alphaherpesvirus 1 (FHV-1), affects replication of each agent in this cell culture system. We also investigated the metabolic impact of each co-pathogen using metabolomic analysis of infected and coinfected cells. *C. felis* was able to invade and replicate in CRFKs, while *M. felis* had little capacity to invade. During coinfection, FHV-1 replication was minimally affected by the presence of either bacterial pathogen, but bacterial replication kinetics were more affected, particularly in *M. felis*. Both *C. felis* and *M. felis* replicated to higher levels in the presence of a secondary pathogen. Coinfections resulted in reprogramming of the glycolysis pathway, the pentose phosphate pathway, and the tricarboxylic acid cycle. The distinct metabolic profiles of coinfected cells compared to those of cells infected with just one of these three pathogens, as well as the impact of coinfections on viral or bacterial load, suggest strong interactions between these three pathogens and possible synergistic mechanisms enhancing virulence that need further investigation.

**IMPORTANCE:**

In the natural world, respiratory pathogens coexist within their hosts, but their dynamics and interactions remain largely unexplored. Herpesviruses, mycoplasmas, and chlamydias are common and significant causes of acute and chronic respiratory and system disease in animals and people, and these diseases are increasingly found to be polymicrobial. This study investigates how coinfection of feline cells between three respiratory pathogens of cats impact each other as well as the host innate metabolic response to infection. Each of these pathogens have been implicated in the induction of feline upper respiratory tract disease in cats, which is the leading cause of euthanasia in shelters. Understanding how coinfection impacts co-pathogenesis and host responses is critical for improving disease management.

## INTRODUCTION

It is well recognized that respiratory tract disease in both animals and humans is often a consequence of simultaneous infections with multiple pathogens, which can exacerbate disease through a range of different mechanisms. Feline upper respiratory tract disease (URTD) in cats is the leading cause of euthanasia in shelters and is commonly caused by one or a combination of felid alphaherpesvirus 1 (FHV-1), feline calicivirus (FCV), *Bordetella bronchiseptica*, *Chlamydia felis*, and *Mycoplasma felis* (1–5). *M. felis*, FHV-1, and *C. felis* are endemic in cats, particularly in shelter populations, and are often associated with respiratory, neurological, and reproductive tract diseases (6, 7). Their ability to establish chronic or life-long infections in an animal enhances opportunities for concurrent infection with multiple pathogens (2). Vaccination against some of these pathogens is used to help control disease, with both inactivated and live-attenuated vaccines available against FHV-1 and *C. felis*, but immunity conferred by these vaccines is not sterilizing; they can protect against severe clinical disease and mortality, but not from infection and transmission (8–10).

Epidemiological associations between herpesviruses and several bacterial species, including *Chlamydia* and *Mycoplasma* spp., have been identified in diverse hosts, including cattle, humans, koalas, cats, and crocodiles (11–16). A study investigating the connection between bacterial ocular surface microbiota and outcomes in cats with FHV-1 ocular surface disease revealed that, while coinfection with *M. felis* had the highest prevalence, coinfection with *C. felis* was significantly associated with worse disease outcomes (17). In cattle, coinfections with herpesviruses and mycoplasmas can exacerbate disease induced by other pathogens. In addition, higher bacterial and viral loads, and reduced antibody responses to herpesvirus infection, have been observed in coinfected animals (15). Similarly*,* coinfections of koalas with *Chlamydia pecorum* and koala herpesviruses were associated with an increased incidence of urogenital disease (16, 18). Coinfection of the respiratory tract of cats with viral pathogens, particularly FHV-1 and FCV, is commonly reported. The prevalence of detection of coinfections is often attributed to a synergistic effect between the co-pathogens, which may arise from FHV-1-induced airway damage, potentially facilitating secondary FCV infection by compromising mucociliary clearance and immune defenses (19, 20).

Herpesviruses, mycoplasmas and chlamydias are capable of invading host cells, often infecting the same types of cells, resulting in upper respiratory tract infections *in vivo*, and can evade or modulate host immune responses (21–23). Intracellular pathogens depend on host energy and biosynthetic pathways for their own replication and can manipulate the metabolism of host cells (24–28). However, the effect of viral–bacterial coinfection on cellular metabolism has not been thoroughly investigated, and it is unclear if metabolic programming of host cells by the primary pathogen affects the secondary infection. *In vitro* coinfection assays using human chlamydial and herpesviral pathogens showed that secondary infection with a herpesvirus arrested the bacterial lifecycle, resulting in increased bacterial persistence within the cell (29). In addition, prior infection of cells with chlamydias affected virus activity, allowing virus entry into cells that were normally not susceptible to infection (30). These viral–bacterial coinfection studies with human pathogens found that these interactions probably involved distinct metabolic mechanisms, such as increased oxidative stress (herpesvirus/chlamydia) (29) and depletion of arginine and sugars (herpesvirus/mycoplasma) (31). While cats naturally infected with *Bartonella henselae* often remain asymptomatic, the presence of concurrent feline immunodeficiency virus (FIV) infection has been linked with the development of clinical signs. Specifically, cats coinfected with both pathogens exhibit a heightened prevalence of antibodies against both pathogens, potentially contributing to the manifestation of clinical characteristics such as gingivitis and lymphadenopathy (32). While coinfections are known to play an important role in disease progression and severity more broadly, and coinfections are common in cats, no coinfection studies have investigated the *in vitro* dynamics between feline herpesviruses and bacterial pathogens. Thus, the interactions between these different pathogens, including any synergistic mechanisms that may enhance virulence, remain undescribed.

Currently, there is a lack of feline respiratory cell lines available for conducting *in vitro* experiments focused on feline pathogens. The alternative approach would be using primary cell cultures, which would be challenging due to their heterogeneous nature, hindering global reproducibility. In contrast, Crandall–Rees feline kidney (CRFK) cells are a globally available and well-established feline epithelial-type cell line (33), and have been used extensively as a reliable model for respiratory virus research (34–36). They are known to be permissive for replication of several intracellular feline pathogens associated with feline respiratory tract diseases, including FHV and FCV (37–39). To date, no *in vitro* coinfection studies of feline pathogens have been undertaken, but the availability of CRFK cells worldwide ensures the reproducibility of experiments across scientific communities and offers a convenient *in vitro* model that can be adapted to study diverse coinfection partners.

In this study, we aimed to investigate how coinfection of feline cells with three common viral and bacterial respiratory pathogens of cats, FHV-1, *C. felis,* and *M. felis*, may affect the replication dynamics of these pathogens and metabolic profiles of the infected cells.

## MATERIALS AND METHODS

### Cells, viruses, and bacterial strains

The FHV-1 strain used in this study was the Companion vaccine F2-like strain commonly used globally, including in Australia (40). Viral stocks were cultured in CRFK cells as described previously (40). The *C. felis* (strain WB96) was initially isolated in cycloheximide-treated McCoy cells from a clinical case of feline URTD in 1996 in Victoria, Australia (41). The *M. felis* (strain 329) used in this study was also isolated from a case of feline URTD. Initially, species confirmation was achieved by sequencing the 16S rRNA gene. Subsequently, the whole-genome sequence was characterized using hybrid assembly methods (42). The *M. felis* strain was cultured in Mycoplasma Broth (MB) containing 10% swine serum (Sigma-Australia) and 0.01% nicotinamide adenine dinucleotide (Sigma-Australia), a minor modification of Frey’s medium (43, 44). The mycoplasma cultures were incubated at 37°C for 18 h. All *in vitro* coinfection assays were performed in CRFK cells as described below.

### Quantification of felid alphaherpesvirus 1 and *Chlamydia felis* by quantitative PCR (qPCR)

Chlamydial and herpesvirus titers were determined using qPCR assays optimized in prior studies (45–48) to determine the relative genome copy concentrations in each sample. Many of the antibiotics used to inhibit chlamydia are bacteriostatic, not bactericidal, and would likely not sufficiently block bacterial contamination of cells during FHV-1 titration of live virus. In particular, antibiotics would not block chlamydial elementary bodies (which are metabolically inert and difficult to eradicate) during FHV-1 titration. Additionally, there were no means to specifically block FHV-1 contamination of cells when attempting to independently titrate *C. felis* via infectivity assays. Given these limitations, titrations relied on genome copy number (gcn) determination as a preferable alternative for both of these pathogens. The FHV-1 qPCR used in this study targeted the FHV-1 thymidine kinase (TK) gene and produced a product of 75 bp (46). Three microliters of extracted DNA was used as the template, with 170 nM of each primer (TK_fwd 5′-CAGTGTTTTCAAAGCCCGGG; TK_rev 5′-GCGGCGGCACATTCATC). Each 20 µL reaction also contained 200 µM of each deoxynucleoside triphosphate (dNTP), 1 mM MgCl_2_, 5 × GoTaq Colorless Flexi buffer, 8 µM of Syto9, and 1 U of GoTaq Flexi DNA polymerase (Promega, USA). Samples were incubated at 95°C for 3 min, followed by 35 cycles of 95°C for 30 s, 58°C for 30 s, and 72°C for 20 s. The chlamydia 16SG qPCR assay used in our studies is commonly used for diagnostic purposes and targets the 16S rRNA gene of *Chlamydiales* (45, 47, 48). Similarly, 3 µL of extracted DNA was used as the template, with 2 µM of each primer (Fwd 5′-TGATGAGGCATGCAAGTC; Rev 5′-TTACCTGGTACGCTCAAAT). Each 20 µL reaction also contained 200 µM of each deoxynucleoside triphosphate (dNTP), 2 mM MgCl_2_, 5 × GoTaq Colorless Flexi buffer, 8 µM of Syto9, and 0.24 µL of GoTaq Flexi DNA polymerase (Promega, USA). Samples were incubated at 95°C for 3 min, followed by 35 cycles of 96°C for 20 s, 58°C for 20 s, and 72°C for 25 s, followed by 72°C for 2 min. A standard curve was employed for each qPCR assay, composed of 10-fold serial dilutions of purified plasmids, in triplicate or duplicate, containing either the TK or 16SG gene sequences cloned into the plasmid vector pGEMT-Easy (46, 47). These standards were performed to determine the relative gcn/mL using an AriaMx Real-Time PCR System (Agilent Technologies).

### Chlamydial infection of CRFK cells

Previous studies have usually cultured *C. felis* in Vero or McCoy cells, which were derived from an African green monkey and a mouse, respectively. To determine if the *C. felis* isolate was capable of infecting and invading CRFK cells, sub-confluent monolayers were inoculated in triplicate with strain WB96, and cultures were centrifuged at 500 × *g* at room temperature for 30 min. Cultures were then incubated for 4 h at 37°C in a humidified 5% CO_2_ incubator. The medium (0.5% v/v fetal bovine serum in Dulbecco’s Modified Eagle Medium [DMEM], 1 mM HEPES pH 7.4) was then supplemented with cycloheximide at a concentration of 1 µg/mL (which is commonly used to inhibit host cell protein synthesis without inhibiting chlamydial protein synthesis), and the cultures were incubated for up to 4 days post infection (DPI). To determine if infection and replication in CRFKs can be established in the absence of cycloheximide, cultures were also incubated in medium without cycloheximide. Samples were frozen at −80°C every 24 h for up to 4 days, and DNA was extracted from the cultures using the MagMAX™ CORE Nucleic Acid Purification Kit (ThermoFisher Scientific). Invasion and replication in CRFK cells were measured using a quantitative PCR (qPCR) targeting the bacterial 16S rRNA gene as described above, and by Diff-Quik (Polysciences) staining of infected cells at 3 DPI.

### Mycoplasmal co-culture with CRFK cells

To detect growth of *M. felis* in CRFK cells, a fresh culture in MB was obtained after 18 h of incubation at 37°C. Mycoplasmas were harvested by centrifugation (17,000 × *g* for 5 min at 4°C) and washed in ice-cold phosphate buffered saline (PBS) (17,000 × *g*, 5 min, 0°C). The pellet was then resuspended and diluted in cell culture maintenance medium (0.5% v/v fetal bovine serum in DMEM, 1 mM HEPES pH 7.4), and 500 µL of the mycoplasmas were inoculated in triplicate onto sub-confluent monolayers of CRFK cells in 12-well plates at a multiplicity of infection (MOI) of approximately 0.03, or into cell culture maintenance medium at the same concentration. The cultures were then incubated at 37°C. After 1 h of incubation, 500 µL of fresh cell culture maintenance medium was added to each culture, and the culture was then incubated at 37°C. Samples were collected and frozen at −70°C every 24 h for 3 days for further titration. The concentration of *M. felis* was determined by counting colonies on inoculated sheep blood agar (SBA) plates (in triplicate) after 7 days of incubation, as described previously (49).

### Mycoplasmal invasion of CRFK cells

To investigate the capacity of *M. felis* to invade CRFK cells, a fresh pellet was prepared as described above. The *M. felis* pellet was resuspended and diluted in the cell culture maintenance medium and inoculated in triplicate onto sub-confluent monolayers of CRFKs at an MOI of approximately 2 or 20, and the cultures were then incubated at 37°C. The infected cells were washed with PBS and trypsinized after 1, 2, and 3 days of incubation. The cells were then harvested and incubated in 500 µL of fresh cell culture maintenance medium containing 400 µg gentamicin/mL at 37°C for 3 h. The CRFK cells were then pelleted by centrifugation at 1,500 × *g* for 5 min, washed with PBS, and resuspended in 500 µL of MB. Viable mycoplasma concentrations were detected by inoculation of SBA plates in triplicate and enumeration of colonies after incubation for 7 days.

### Coinfection of CRFK cells with FHV-1, *C. felis*, and *M. felis*

**Fig 1:**
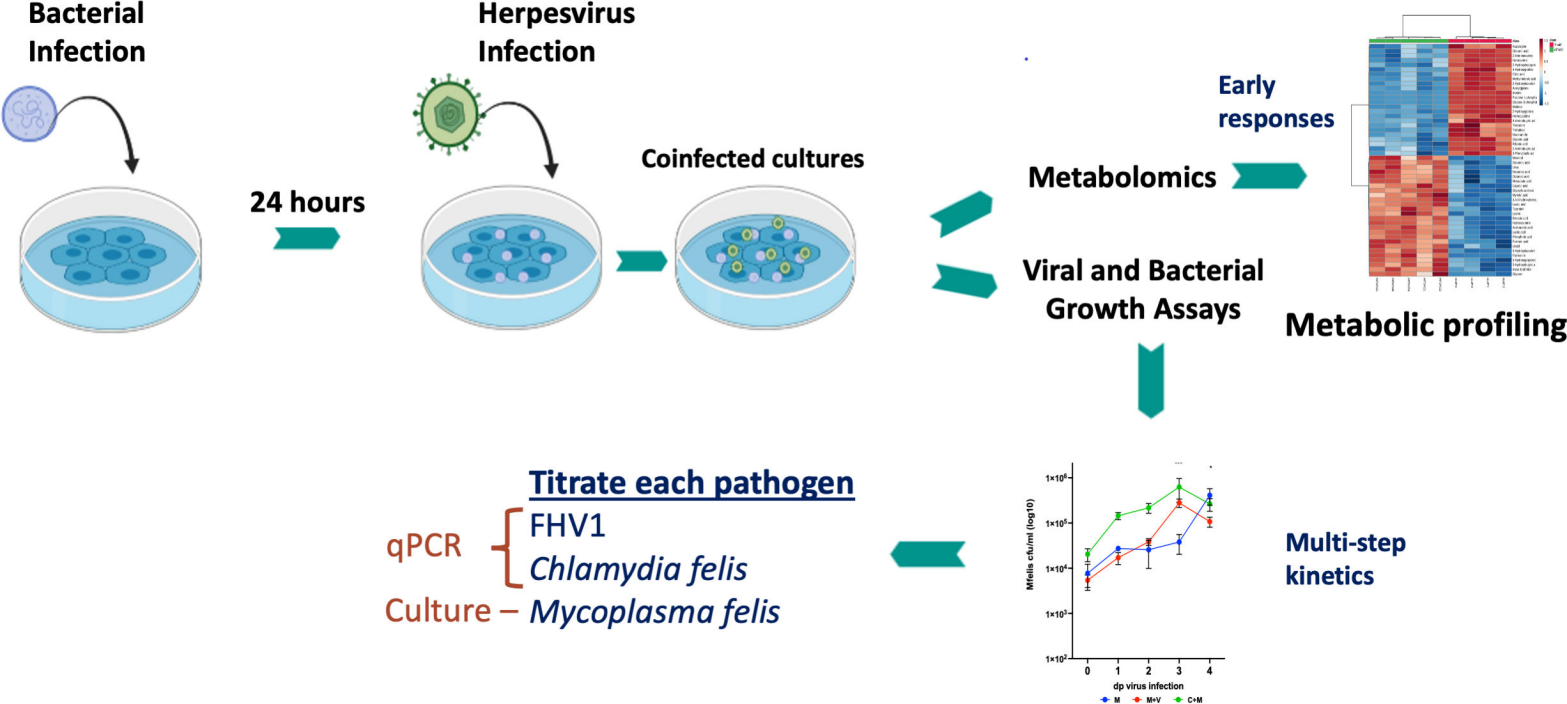
*In vitro* coinfection experiments performed in this study. Feline cells (CRFKs) were inoculated with *C. felis* and/or *M. felis* and incubated for 24 h. The next day, cultures were either mock inoculated or inoculated with FHV-1. Cultures were analyzed to assess viral–bacterial infection dynamics over several days in a multi-step growth assay, and early metabolic profiles at 4 h after viral infection and 1 day post bacterial infection. Figure partially created with BioRender.

A coinfection assay was performed to compare how prior or simultaneous exposure to a second co-pathogen influences the growth kinetics in cell culture of each of the co-pathogens over time (Fig. 1). Sub-confluent monolayers of CRFK cells were inoculated in triplicate with *M. felis* or *C. felis* at an MOI of approximately 1. Infected and mock-infected CRFK cell cultures were incubatedat 37°C for 24 h as described above for each of the pathogens, after which the inoculum was removed, and the cells were gently washed with PBS to remove any bacteria that had not adhered or invaded. FHV-1 or maintenance medium was applied to cultures at a viral MOI of approximately 0.001 and cultures were maintained for up to 4 days in a humidified incubator with 5% CO_2_. At each daily timepoint (0–4 days after viral infection), samples were frozen and stored at −70°C. Upon thawing, mycoplasma concentrations were determined by plating in triplicate onto SBA. DNA was extracted from a 150 µL sample of each culture using a MagMax core kit, and herpesviral and chlamydial titers determined as described above.

### Metabolomic analyses

A total of 42 biological replicates of sub-confluent monolayers of CRFKs were prepared in 6-well plates. Four groups were inoculated with either *M. felis* at an MOI of approximately 1 (12 biological replicates), *C. felis* at an MOI of approximately 1 (12 biological replicates), both bacteria at an MOI of approximately 1 for each bacterial species (six biological replicates), or maintenance medium (12 biological replicates). After 24 h of incubation at 37°C in 5% CO_2_ in air in a humidified incubator, the cells were washed with PBS to remove bacteria that had not adhered to or invaded into the CRFK cells. Maintenance medium was then added to six biological replicates from each inoculated group: *M. felis* single infection (M), *C. felis* single infection (C), bacterial coinfection (M + C), and uninfected (Mock). FHV-1, at an MOI of approximately 12, was inoculated into the remaining six biological replicates that were initially inoculated with *M. felis*, *C. felis*, or maintenance medium to generate CRFK cultures coinfected with FHV-1 and *M. felis* (M + FHV), coinfected with FHV-1 and *C. felis* (C + FHV), or infected with only FHV-1 (FHV), respectively. An MOI of 12 was used for the metabolomic studies to ensure synchronized infection of all CRFK cells by FHV-1. All the cultures were incubated for a further 1 h at 37°C in 5% CO_2_ in air in a humidified incubator. The cells were then washed twice with PBS to remove virus that had not adhered or been internalized. All the cultures were further incubated for a further 3 h at 37°C in 5% CO_2_ in air in a humidified incubator. The brief incubation period of 4 h with FHV-1 ensured that the infected cells were typically in an early stage of viral infection and had not yet undergone apoptosis, which could potentially confound metabolomic profiling results. The cells were then washed twice with PBS to remove media residues, and the PBS was completely aspirated from the wells, followed by a gentle wash with pre-warmed distilled sterile water, to reduce phosphate contamination. To halt metabolic activity within the samples, the water was completely aspirated from the cultures and liquid nitrogen was added to the wells to quench the cells. The cells were immediately stored at −70°C on dry ice until further processing. The polar metabolites were extracted and derivatized as described previously (50), then analyzed on a gas chromatography-mass spectrometry (GC-MS) system comprising an AOC6000 autosampler, a 2030 Shimadzu gas chromatograph, and a TQ8050NX triple quadrupole mass spectrometer (Shimadzu, Japan), as described previously (51).

### Statistical analysis

Viral and bacterial replication dynamics in coinfected cells were analyzed using multiple Holm–Sidak corrected *t*-test comparisons to singly infected controls, using the Holm–Sidak method of *P*-value correction in Graphpad Prism v 9.0. The statistical analysis for metabolomic experiments was performed using Graphpad Prism v 9.5 and MetaboAnalyst (52). The data matrices obtained from the GC-MS analyses were normalized by median and log transformation, after the removal of the two outlier replicates from each group, using MetaboAnalyst. Viral and bacterial coinfection metabolomics were analyzed by multiple *t*-test comparisons to singly infected or mock-inoculated controls, using the Benjamini and Hochberg false discovery rate (FDR) approach, with the FDR adjusted *P* value < 0.05 regarded significant. The effects of infections on metabolism were examined by comparison of the metabolite profiles of each singly and coinfected group to that of the mock-inoculated group. To evaluate the effect of FHV-1 and *M. felis* coinfections on metabolism, the metabolite profile of the M + FHV group was also compared to those of the groups singly infected with FHV-1 or *M. felis*. Similarly, the metabolite profile of the C + FHV group was also compared to those of the FHV-1 or *C. felis* single infection groups. Finally, the metabolite profile of the bacterial coinfection treatment group (M + C) was compared to the groups singly infected with either *M. felis* or *C. felis*.

## RESULTS

### *Chlamydia felis* was able to invade and replicate in feline CRFK cells in the presence and absence of cycloheximide

**Fig 2:**
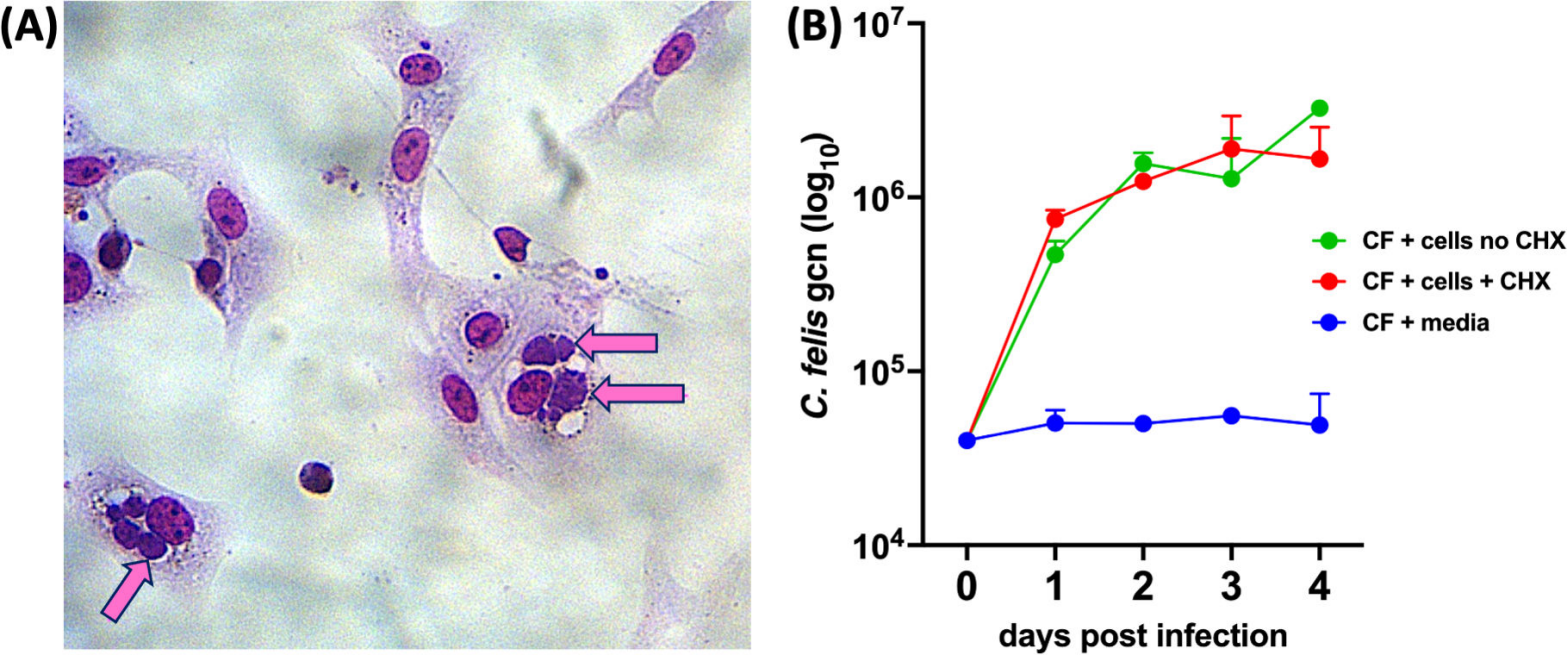
**(A**) CRFK cells infected with *C. felis* and stained with Diff-quick (×320 magnification). Arrows indicate large *C. felis* clusters (stained purple) within the cells. (**B**) *C. felis* infection of CRFK cells over 4 days after infection, in the presence or absence of cycloheximide (CHX). CF, *C. felis*; CHX, cycloheximide; gcn/mL, genome copy numbers. Data are shown as mean ± standard deviation.

*Chlamydia felis* strain WB96 was found to be able to invade and replicate in CRFKs over 4 DPI to titers of approximately 3.16 × 10^6^ genome copy numbers per millilitre (gcn/mL) (Fig. 2). The presence or absence of cycloheximide did not significantly impede replication, and rapid replication over the 4 days also occurred in samples from which the inoculum was removed at 4 h after infection.

### *Mycoplasma felis* was able to replicate in the presence of CRFK cells, but only a low level of invasion was observed

**Fig 3:**
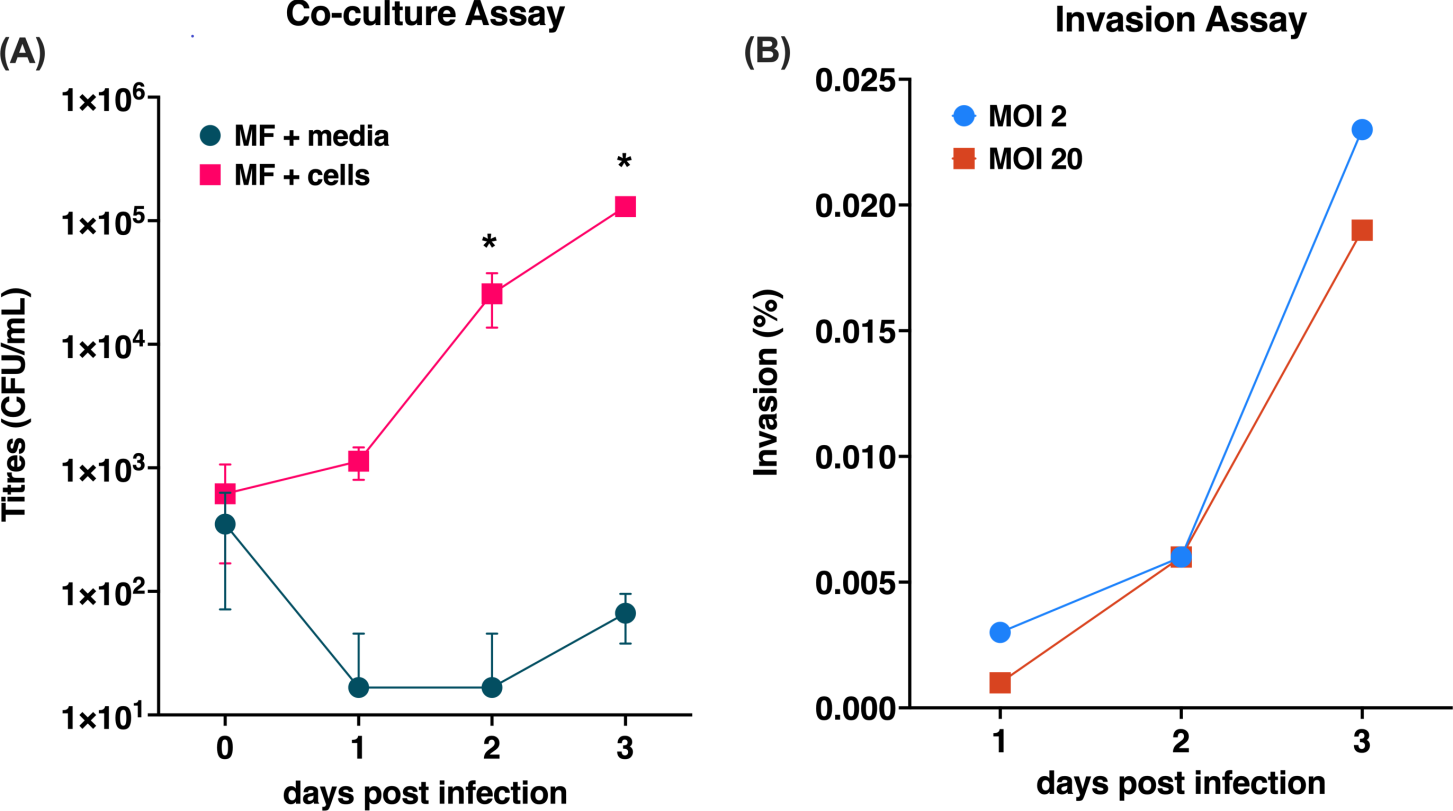
*Mycoplasma felis* (MF) *in vitro* culture assays. (**A**) Co-culture assay showing that replication of *M. felis* is supported in the presence of CRFK cells but not in cell culture medium only; data are shown as mean ± standard deviation. *P < 0.05 (Multiple Holm–Sidak corrected *t*-test). (**B**) Gentamicin invasion assay assessing entry of *M. felis* into CRFK cells, indicating that MF has a low capacity to enter CRFK cells over 3 days of infection. Media, DMEM; CFU/mL, colony-forming units per milliliter; MOI, multiplicity of infection.

The co-culture assay showed that *M. felis* was able to replicate in CRFK cell cultures over 3 DPI to titers of up to 1.2 × 10^5^ colony-forming units (CFU)/mL (Fig. 3A). Comparison of *M. felis* replication in CRFK cell cultures to replication in cell culture medium only showed that significantly higher titers were detected in the presence of CRFK cells at 2 (*P* < 0.01) or 3 (*P* < 0.0001) days after infection (Fig. 3A). Invasion of the CRFK cells by *M. felis* was negligible, with only approximately 0.02% of the bacterial inoculum internalized into CRFK cells by 3 DPI (Fig. 3B).

### Coinfection with FHV-1 or *C. felis* significantly increased mycoplasma replication in CRFK cells

**Fig 4:**
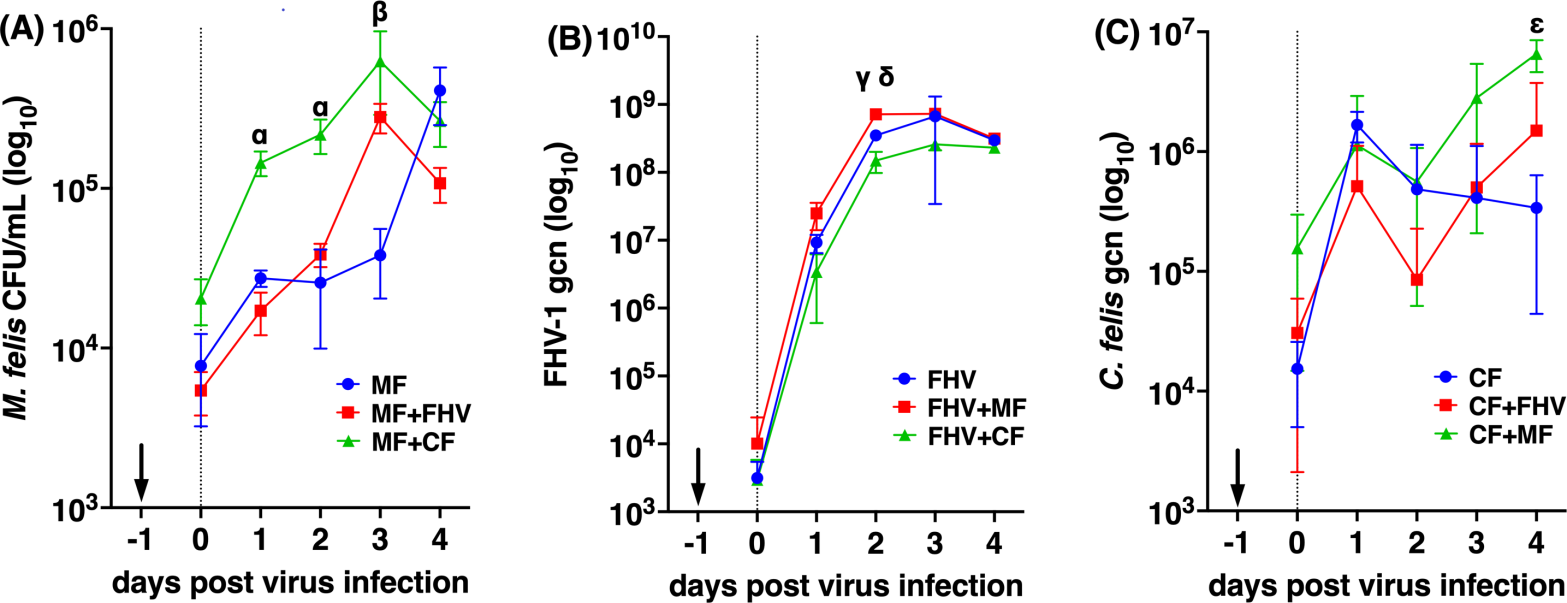
Coinfection of CRFK cells with three feline URTD pathogens, *M. felis* (MF), *C. felis* (CF), and felid alphaherpesvirus 1 (FHV-1) over 4 days, showing the impact of co-culture on bacterial and viral replication kinetics. Arrows indicate infection of CRFK cells with bacterial species 1 day prior to FHV-1 infection. (**A**) *M. felis* growth curves, (**B**) FHV-1 replication curves, and (C) *C. felis* growth curves. Data are shown as mean ± standard deviation. Significantly different titers of each pathogen, comparing singly infected to coinfection groups are indicated (multiple Holm–Sidak corrected *t*-tests); *M. felis* in coinfections with *C. felis* (MF +CF; α, *P* < 0.01) or FHV-1 (MF +FHV; β, *P* < 0.01) compared to singly infected (MF) cells; FHV-1 genome copy numbers (gcn/mL) in cells coinfected with *C. felis* (FHV + CF; γ, *P* < 0.01) or *M. felis* (FHV + MF; δ, *P* < 0.001) compared to singly infected (FHV1) ; and *C. felis* gcn/mL in cells coinfected with *M. felis* (CF +MF; ε, *P* < 0.01) compared to singly infected (CF) cells; gcn/mL, genome copy numbers per milliliter; CFU/mL, colony-forming units per milliliter.

The impact of coinfection on *M. felis* replication was assessed by performing colony counts on sheep blood agar (Fig. 4A). Coinfection with FHV-1 was delayed, with virus inoculated 1 day after bacterial infection of the cells (Fig. 4A, Day 0), while *M. felis* and *C. felis* were inoculated simultaneously (Fig. 4A, Day −1). Coinfection with C. *felis* resulted in a significant increase in *M. felis* replication at Days 1 and 2, while coinfection with FHV-1 resulted in a significant increase in *M. felis* replication only at Day 3. *M. felis* titers declined in the coinfected cultures by Day 4 , but continued to increase in the absence of any co-pathogen, although this difference was not significant.

### Coinfection with *M. felis* or *C. felis* had minimal impact on FHV-1 replication *in vitro*

The impact of coinfection with a mycoplasmal or a chlamydial pathogen on FHV-1 replication over time was assessed in a multi-step growth curve, with viral genome copy concentrations determined using an FHV-1-specific qPCR. Coinfection with either bacterial pathogen had only minor impacts on viral replication, with significant differences (*P* < 0.05) only at 2 days post viral infection (Fig. 4B). Viral titers in cells previously infected with *M. felis* were two times higher at this timepoint than in cells only infected with the virus (*P* < 0.001). In contrast, virus titers in cells previously infected with *C*. *felis* were 57.4% lower at this timepoint than in cells only infected with FHV-1 (*P* = 0.013). From Day 3 onward, viral titers were similar regardless of coinfection status.

### Coinfection with FHV-1 had no impact on chlamydial DNA replication, but coinfection with *M. felis* enhanced *C. felis* replication

The impact of coinfection on chlamydial gcn concentrations (as a proxy for bacterial replication) was assessed using qPCR (Fig. 4C). As was described above for the mycoplasma coinfections, secondary coinfection with FHV-1 was performed by inoculating the CRFK cells 1 day after bacterial infection (Fig. 4C, Day 0). No significant difference was detected in chlamydial gcn concentrations when cells were coinfected with *C. felis* and FHV-1, but significantly higher chlamydial gcn were detected at Day 4 when CRFK cells were coinfected with *C. felis* and *M. felis* (*P* = 0.027).

### Coinfections had a major impact on catabolic (energy generating) pathways in host cells

The catabolic pathways facilitate transformation of initial substrates, such as carbohydrates or proteins, into metabolites that are able to enter glycolysis, the tricarboxylic acid (TCA) cycle, or the pentose phosphate pathway (PPP). These three pathways collectively form the core of “central metabolism,” as they serve as critical intersections for a wide range of metabolic pathways (53).

**Fig 5:**
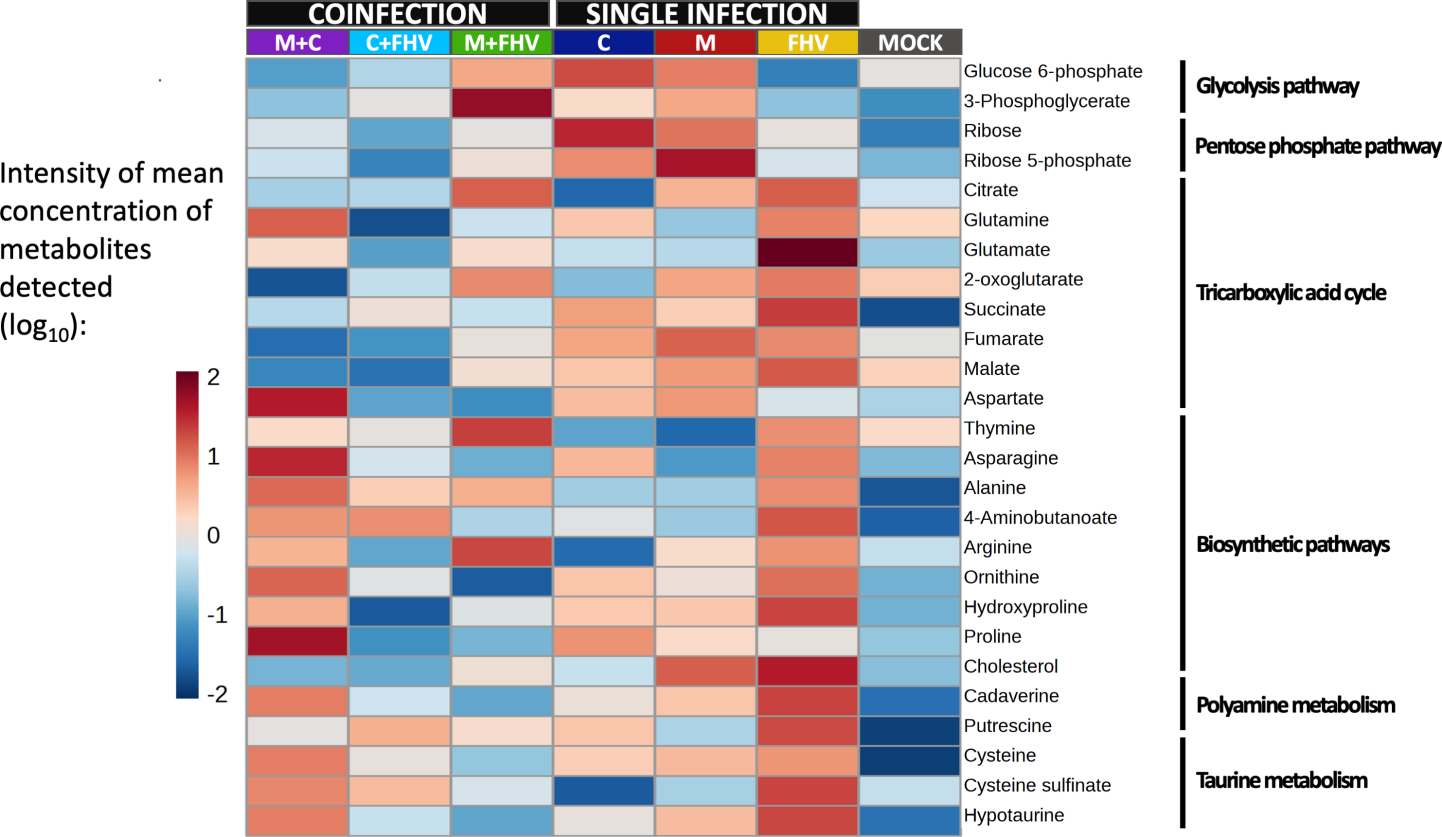
Heatmap showing the intensity of the mean concentration of the metabolites detected in infected cells that differed significantly in abundance between at least two groups (single infections and coinfections compared to mock inoculated, and coinfections compared to each single infection) (Benjamini and Hochberg corrected *t*-test; false discovery rate [FDR] adjusted *P* value < 0.05). The data are median normalized and log_10_ transformed. Mock, inoculation with medium; FHV, single infection with FHV-1; M, single infection with *M. felis*; C, single infection with *C. felis*; M + FHV, coinfection with *M. felis* and FHV-1; C + FHV, coinfection with *C. felis* and FHV-1; M + C, coinfection with *M. felis* and *C. felis*.

**Fig 6:**
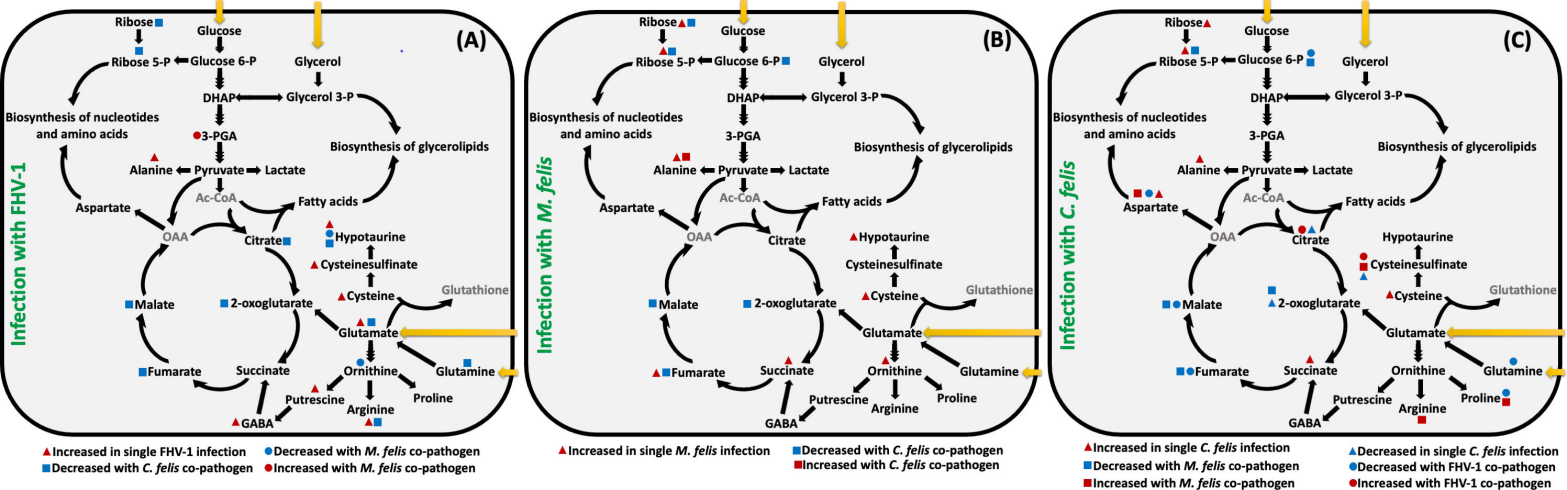
Schematic overview of significant effects on cellular metabolism of (A) single or coinfection with FHV-1; (**B**) single or coinfection with *M. felis*; and (C) single or coinfections with *C. felis* (Benjamini and Hochberg corrected *t*-tests; false discovery rate [FDR] adjusted *P* value < 0.05). Yellow arrows indicate metabolite uptake from medium. Metabolites in gray were not detected in our study. DHAP, dihydroxyacetone phosphate; Ac-CoA, acetyl CoA; OAA, oxaloacetic acid; GABA, 4-aminobutanoate; -P, -phosphate.

A total of 139 metabolites were detected in CRFK cells, as outlined in Table S1. The significantly perturbed metabolite concentrations (*P* < 0.05) within the impacted pathways are summarized in Fig. 5 and 6. The effect of infections on metabolism were examined by comparing the metabolite profiles of different groups of singly and coinfected cells to the metabolite profile of the mock-inoculated group. In addition, the impact of FHV-1 and *M. felis* (M + FHV) coinfection, FHV-1 and *C. felis* (C + FHV) coinfection, and bacterial coinfection (M + C) on metabolic dynamics were evaluated separately by comparing the metabolite profiles of the coinfected groups to those of the singly infected groups.

No significant difference was detected in glycolysis intermediates of cells infected with only one pathogen, but bacterial coinfection (M + C) resulted in a significantly lower abundance of glucose-6-phosphate compared to cells infected with either *M. felis* or *C. felis* alone (Fig. 5). Similarly, coinfection with FHV-1 and *C. felis* resulted in significantly reduced abundance of glucose-6-phosphate in CRFK cells compared to infection with *C. felis* alone. Cells coinfected with FHV-1 and *M. felis* had a significantly higher abundance of 3-phosphoglycerate compared to cells that were mock-infected or infected with FHV-1 alone.

Within the pentose phosphate pathway, two metabolites were significantly impacted by infection. The abundances of ribose and ribose 5-phosphate were significantly higher in CRFK cells infected with either of the bacterial pathogens alone (Fig. 5). However, coinfection with FHV-1 and *C. felis* resulted in a significantly lower abundance of ribose and ribose 5-phosphate than in cells infected with *C. felis* alone. Similarly, cells coinfected with both bacterial pathogens had significantly lower levels of ribose than those infected with either bacterial species alone, and a lower ribose 5-phosphate abundance than cells infected with *M. felis* alone.

A major impact was observed on the TCA cycle of infected cells (Fig. 5). Infection of CRFK cells with FHV-1 alone resulted in a significantly higher abundance of glutamate compared to mock-inoculated cells. Cells infected with *M. felis* had significantly elevated concentrations of fumarate and succinate than mock-inoculated cells. Conversely, in cells infected with *C. felis*, the abundances of citrate and 2-oxoglutarate levels were significantly lower, and those of succinate and aspartate significantly higher, than in mock-inoculated cells. The abundances of citrate, fumarate, malate, 2-oxoglutarate, glutamine, and glutamate were significantly lower in FHV-1- and *C. felis*-coinfected cells than in cells infected with FHV-1 alone. Significantly lower abundances of fumarate, malate, and 2-oxoglutarate were detected in bacterial coinfected cells (M + C) than in cells infected with *M. felis* alone, and significantly increased abundance of aspartate and decreased abundances of fumarate, malate, and 2-oxoglutarate compared to mock-inoculated cells. The abundances of glutamine, fumarate, malate, and aspartate were significantly lower, and the abundance of citrate was significantly higher in the cells coinfected with FHV-1 and *C. felis* than in cells infected with *C. felis* alone.

### Coinfections altered biosynthetic pathways in host cells

Central cellular metabolism also generates intermediate molecules that serve as essential components for anabolic pathways, such as those for proteins (constructed from amino acid monomers), lipids (composed of fatty acids monomers), and DNA molecules (composed of nucleotides monomers).

Infection with FHV-1 alone resulted in significantly higher abundances of asparagine, alanine, 4-aminobutanoate (GABA), arginine, and hydroxyproline. Infection with *M. felis* alone resulted in significantly higher abundance of alanine, and infection with *C. felis* alone resulted in higher abundances of asparagine, alanine, ornithine, proline, and hydroxyproline and a significantly lower abundance of thymine (Fig. 5). A significantly higher abundance of alanine was detected in the cells coinfected with FHV-1 and *C. felis*, and FHV-1 and *M. felis*, compared to mock-inoculated cells. Coinfection with FHV-1 and *C. felis* resulted in a significantly lower abundance of hydroxyproline than in cells infected with *C. felis* or FHV-1 alone, and significantly lower abundance of proline than in mock-inoculated cells or cells infected with only *C. felis* and a lower abundance of arginine than in cells infected with FHV-1 alone. The cells coinfected with FHV-1 and *M. felis* had significantly lower abundances of ornithine and asparagine than cells infected with FHV-1 alone. Comparisons of the bacterial coinfected (M + C) cells to cells that were mock inoculated or infected only with *M. felis* revealed significantly higher abundances of proline, asparagine, alanine, and 4-aminobutanoate (GABA). The abundances of arginine and proline were significantly higher in cells coinfected with the two bacterial pathogens than in the cells infected only with *C. felis*.

No significant changes in polyamine metabolism were detected in cells infected with only one of the bacterial pathogens, but infection with FHV-1 alone resulted in significantly higher abundances of cadaverine and putrescine, and bacterial coinfection resulted in significantly higher abundances of cadaverine (Fig. 5). In contrast, the abundance of cadaverine was significantly lower in cells coinfected with FHV-1 and *C. felis* than in cells infected with only FHV-1.

Taurine metabolism was also affected in all the infected cells. Cells infected with only *C. felis* or *M. felis* had an increased abundance of cysteine compared with mock-inoculated cells, while the abundance of cysteine sulfinate was significantly lower in cells infected with *C. felis* alone. Cells infected with *M. felis* or FHV-1 alone had significantly higher levels of hypotaurine than mock-inoculated cells, while coinfection with FHV-1 and either *M. felis* or *C. felis* resulted in significantly lower levels of hypotaurine compared to infection with FHV-1 only. Cells coinfected with *C. felis* and *M. felis* had significantly higher abundances of cysteine, cysteine sulfinate, and hypotaurine compared to mock-inoculated cells. The abundance of cysteine sulfinate was significantly increased in cells coinfected with *C. felis* and either FHV-1 or *M. felis*, compared to cells infected with only *C. felis*.

Infection with FHV-1 significantly increased levels of cholesterol, whereas infection with either of the bacterial pathogens affected glycerolipid metabolism to a lesser extent. In contrast, coinfection with FHV-1 and *C. felis* resulted in significantly lower levels of cholesterol compared to infection with FHV-1 alone.

## DISCUSSION

In this study, we demonstrated that CRFK cells are able to support the replication of both *C. felis* and *M. felis*, in addition to that of FHV-1. The impact of coinfection on pathogen replication dynamics in these cells varied, depending on the pathogens. *M. felis* replication was enhanced by coinfection, while replication of FHV-1 and *C. felis* were less impacted. While the presence of FHV-1 did not significantly influence the replication of *C. felis* within the CRFK cells, cell cultures previously infected with *C. felis* had a significantly lower FHV-1 titer at 2 days post virus infection. While the reduction of FHV-1 titers observed in this study is limited, it suggests a possible suppressive effect of *C. felis* on FHV-1 replication, which aligns with a previous study investigating the prevalence of *C. felis* and FHV-1 in cats with URTD. Both studies suggest that the presence of either pathogen may hinder the replication or detection of the other within the infected host (11). However, replication of FHV-1 and *C. felis* was assessed by measuring DNA replication rather than formation of infectious particles, so it is possible that their infectivity may have been impacted, in particular, that of *C. felis*. *Chlamydia trachomatis* has previously been shown to continue to generate DNA during an arrested replicative stage induced by coinfection with human herpesvirus 1 and 6 (HHV-1 and HHV-6, respectively) (29, 30). Other mycoplasma studies have found a correlation between increased bacterial load and disease severity (54–56). The enhanced replication of *M. felis* under coinfection conditions suggests that *M. felis*-induced disease could also be exacerbated during coinfection as a result of increased mycoplasma load, though no such studies have been performed in cats. While natural infections may occur simultaneously, the order of acquisition and timing of coinfections can influence the host’s immune response, pathogen interactions, and disease severity (57). In our study, we chose to inoculate the cells with bacterial pathogens first, before adding the virus, because inoculating cells with herpesviruses initially could potentially compromise the integrity of the cell monolayer. It is essential to acknowledge that this experimental setup may not fully capture the complexities of all natural coinfection scenarios. Future research exploring alternative orders or simultaneous inoculations could offer valuable insights into how the timing and sequence of coinfections influence disease progression and host responses.

We utilized a steady-state metabolomic approach to provide a snapshot of the CRFK cell metabolic responses at the earlier stages of virus and/or bacterial infection (4 h post virus infection/1 day post bacterial infection) under single or coinfection conditions. At this timepoint, the pathogens exhibited no significant differences in titers (Fig. 4, Day 0), indicating that they were still in the early stages of growth, with titers unaffected by the presence of a co-pathogen. To provide an example, according to the replication assay (Fig. 4A), the viable titer of *M. felis* was less than 10^5^ CFU/mL. Prior studies have demonstrated that a minimum number of 10^7^ mycoplasma cells are needed to detect the metabolome of mycoplasmas on GC/MS (50, 51, 58, 59). For this reason, exploration of the bacterial metabolome is typically conducted either in axenic medium (60) or after mechanical separation of cells and bacteria (28). As a result, the proportion of the metabolome directly contributed by the pathogens in this study is minimal, with profiles based solely on the cell metabolome. We observed that coinfections affected cellular metabolism in differing ways, as summarized in Fig. 6. By examining metabolomic profiles and pathogen replication kinetics, we were able to explore possible connections between metabolic alterations and replication profiles in each of the coinfections.

Coinfection with *M. felis* and *C. felis* is associated with increased severity of URTD, conjunctivitis, and chronic gingivostomatitis in cats (1, 61). While coinfections with mycoplasmas and chlamydias have been reported in humans and other animals (62–66), the cellular mechanisms underlying a possible synergism remain unclear. A recent study showed that vaginal metabolomes of women coinfected with *C. trachomatis* and *Mycoplasma genitalium* were distinct from those of uninfected women and that the vaginal metabolomes of women coinfected with these pathogens were distinct from those of women only infected with *C. trachomatis*, primarily in the lipid biosynthesis pathways, suggesting that infection with the two co-pathogens can have a substantial effect on the metabolome of the host (67). Here, we have shown that coinfection with *C. felis* and *M. felis* affects several metabolites within the glycolysis pathway, the PPP, and the TCA cycle of host cells compared to infection with *M. felis* or *C. felis* alone (Fig. 6). The titers of *M. felis* and *C. felis* were significantly increased under coinfection conditions compared to infection with either of the pathogens alone. The shift of the metabolic state of the CRFKs cells caused by *C. felis*, to anabolism, may have provided nutrients for *M. felis*, enhancing proliferation of this species. Alternatively, increased proliferation of *M. felis* may have been a result of the increased release of intracellular nutrients to the extracellular milieu, where *M. felis* was located, or promotion by *C. felis* of invasion of the CRFK cells by *M. felis* and hence enhanced access to nutrients. Similarly, metabolic perturbations triggered by *M. felis* may have provided energy and/or biomolecules required for replication of *C. felis*. Future immunofluorescence studies are needed to investigate the localization of *M. felis* following coinfection with *C. felis*, as well as impacts of coinfection on the infectivity of *C. felis*.

While little is known about any synergistic relationships between infections with FHV-1 and *M. felis* in cats, in other host species, coinfection with herpesviruses and mycoplasmas can result in more severe disease progression, which is often associated with immunomodulation of the host immune defenses by either viral or bacterial mechanisms (62, 68–76). In cattle, bovine herpesvirus 1 (BHV-1)/*Mycoplasma bovis* coinfections are common contributors to the bovine respiratory disease (BRD) complex. Prior exposure to BHV-1 results in a higher isolation rate of *M. bovis* from coinfected calves 11 days after viral infection, possibly due to viral-induced immune suppression (15). In our study, FHV-1 coinfection promoted more rapid replication of *M. felis* in co-culture, possibly due to destruction of host cells by FHV-1 resulting in release of key metabolites into the extracellular environment. Host immune responses were not directly assessed in our study, but future work to examine possible host immune suppression during FHV-1/*M. felis* coinfections would help to further characterize the relationships between these co-pathogens.

*In vitro* coinfection studies with *Mycoplasma arginini* resulted in reduced titers of HHV-1 in Vero cells, and supplementation of the medium with arginine reversed this effect (31), suggesting a connection between the presence of a secondary pathogen, alterations in host cell metabolism, and viral replication. Here, coinfection with *M. felis* significantly enhanced FHV-1 DNA replication at 2 days post virus infection, although the increase was small. The metabolite profile of the cells coinfected with FHV-1 and *M. felis* was not substantially different from that of control cell cultures infected with only *M. felis*. However, cells coinfected with FHV-1 and *M. felis* resulted in increased abundances of some glycolytic intermediates compared to cells infected with only FHV-1 (Fig. 6). The possible enhancement of glycolysis in cells coinfected with *M. felis* may have provided sufficient energy and/or precursors of biosynthetic pathways for the cells, bacteria, and viruses, and thus improved proliferation of FHV-1. *In vitro* coinfections of primary tissue with mycoplasmas and other viral families have had varying effects, depending on host cell species (77) and perhaps the extent of cytopathic effect induced by each co-pathogen (lysis or silent carriage) (78). Future studies investigating coinfection with *M. felis* and FHV-1 in primary feline tracheal organ cultures, or other primary respiratory organotypic cultures, as well as feline corneal epithelial cells (79), are needed to determine the impact of coinfection on replication kinetics and metabolic perturbations in a more complex biological system.

Chlamydial coinfections with viruses have been reported previously, but their effects on cellular interactions remain unclear (30, 62–66). In our study, FHV-1/*C. felis* coinfection did not alter the replication kinetics or titers of either pathogen substantially, although a small decrease in FHV-1 genome copy numbers was detected at Day 2 in coinfected cells. *In vitro* coinfection with *C. trachomatis* and HHV-1, human cytomegalovirus (HCMV), or HHV-6 has been shown to reduce the production of infectious *C. trachomatis* and increase the viral genome replication of HHV-6, HHV1, and HCMV, but not necessarily increase the production of infectious virus (30). Metabolically, coinfection with HHVs reduced cellular glutathione levels, thereby driving chlamydial persistence, as determined by electron microscopy (29). While glutathione was not detected in our studies, coinfection with *C. felis* and FHV-1 resulted in reduced levels of some of the intermediates of glycolysis and the PPP in cells (Fig. 6). We also observed that coinfection with *C. felis* resulted in depletion of some of the intermediates of the PPP and the TCA cycle at 4 h after virus infection. This could be due to depletion of the cellular levels of intermediates of the PPP and TCA cycle triggered by *C. felis*, and thus reduced available energy and/or biomolecules required for replication of FHV-1. Thus, although only minor differences in the concentrations of FHV-1 and *C. felis* genome copies were seen in this study, future studies to assess infectious titers of both pathogens are indicated and may reveal differences in replication kinetics that were not able to be detected in this study.

In addition to examining relationships between co-pathogens, we also conducted the first study of the metabolism of CRFK cells after infection with three different host-specific pathogens of cats using untargeted metabolomic profiling. This adds to our understanding about the metabolomics of infection with mycoplasmas, chlamydias, and herpesviruses in other host cells. While CRFK cells infected with only one of the two bacterial pathogens had an increase in the abundances of intermediates of glycolysis and the PPP, infection with FHV-1 resulted in only a slight increase in the levels of some of the PPP intermediates (Fig. 6). This is consistent with prior studies in which cells infected with HHV-1 showed increased activity in the glycolytic and pentose phosphate pathways, which is likely to promote viral nucleotide synthesis (80). Many mycoplasma species use glucose as their preferred carbon source, importing glucose and converting it to glucose-6-phosphate (58, 81), while chlamydias typically import glucose-6-phosphate, not glucose, from the host cell into their glycolytic or pentose phosphate pathways and also use it for lipopolysaccharide biosynthesis (82, 83).

The TCA cycle plays a crucial role in providing energy for mammalian cells, and feeds from multiple substrates, including pyruvate or 2-oxoglutarate through glutamate (either via direct glutamate uptake or conversion of glutamine to glutamate). We observed an increase in the abundance of several TCA intermediates and glutamate in CRFK cells infected with FHV-1 alone, without a substantial change in glutamine abundance (Fig. 6). These findings suggest that FHV-1 affects the metabolism of the host cells by increasing influx into the TCA cycle through glycolysis, but not through glutamine, similar to HHV-1 (80). *Chlamydia* spp. encode an incomplete TCA cycle, lacking genes for citrate synthase, aconitase, and isocitrate dehydrogenase, and, therefore, can only be fed by uptake of exogenous glutamate, succinate, fumarate, malate, aspartate, oxaloacetate, or 2-oxoglutarate (82, 84, 85). We found that infection of CRFK cells with *C. felis* significantly reduced the levels of citrate and 2-oxoglutarate, suggesting that the TCA cycle of *C. felis* is likely to be fed via glutamate and 2-oxoglutarate, as has been seen in *C. trachomatis*. Mycoplasmas also lack a functional TCA cycle, although genes encoding lactate/malate dehydrogenase have been reported in other species (58, 86, 87). The presence of *C. felis* with co-pathogens also lacking a functional or complete TCA cycle would be expected to result in even greater appropriation of the host’s TCA intermediates to provide the energy, nucleotides, and amino acids required for bacterial and viral replication, consistent with our findings.

Polyamines, such as putrescine, cadaverine, spermine, and spermidine, are involved in diverse cellular processes in eukaryotes, bacteria, and archaea (88). In mammalian cells, polyamines play a role in protein synthesis, modifying the structure and function of nucleic acids, and altering gene expression (89). Viral replication in mammalian cells depends on the cellular roles of the polyamines, and some viruses upregulate biosynthesis of polyamines in infected cells to utilize them for their own replication (89). While HHV-1 has been shown to enhance uptake of putrescine into infected cells, it also inhibits the production of spermidine and spermine, which are downstream intermediates of polyamine biosynthesis (90). In this study, we observed a significantly higher abundance of putrescine and cadaverine in CRFK cells infected with FHV-1, indicating modulation of the host cell’s polyamine levels after viral infection, consistent with that seen for HHV-1. In other herpesviruses, polyamine metabolism appears to be essential for replication (91, 92). HHV-1 encapsidates polyamines to facilitate genome packaging by neutralizing its negative charge (93), and putrescine, spermine, and spermidine promote HHV-1 DNA polymerase activity at low ionic strength, while high concentrations of spermine inhibit polymerase activity (94). In this study, bacterial infection also resulted in a slight increase in the abundance of the polyamines, albeit not significantly, consistent with prior mycoplasma and chlamydial studies (95, 96).

Feline cells have limited capacity for taurine biosynthesis, and as a result, taurine is considered an essential amino acid for cats (97, 98). In this study, taurine, which has a proposed role in osmoregulation of cells infected with HHV-1 or HCMV (80), was not detected. Instead, CRFK cells infected with FHV-1 had a significant increase in the levels of intermediates of taurine metabolism: cysteine, cysteine sulfinate, and hypotaurine (Fig. 6). Taurine is synthesized from hypotaurine, which is produced from cysteine sulfinate, a resultant of cysteine oxidation. While little is known about the significance of hypotaurine, it is proposed to play a role as a cellular antioxidant (99, 100). Our finding suggests that CRFK cells are capable of biosynthesis of hypotaurine, which is found in the testes or epididymides of cats (101), and that hypotaurine biosynthesis is substantially affected by infection with FHV-1 and *M. felis*.

This study has initiated investigations into the *in vitro* replication dynamics and metabolomics of three important feline co-pathogens. Such investigations are essential for understanding the synergistic relationships between pathogens and ultimately important for controlling the diseases they cause.

## Data Availability

The GC/MS metabolite concentration data used in this study are provided as supplemental material (Table S1).

## SUPPLEMENTAL MATERIAL

Table S1 msystems.00852-24-s0001.docx Metabolite profile data set.

ASM does not own the copyrights to Supplemental Material that may be linked to, or accessed through, an article. The authors have granted ASM a non-exclusive, world-wide license to publish the Supplemental Material files. Please contact the corresponding author directly for reuse.
