# Supplementary material for: A “plus one” strategy impacts replication of felid alphaherpesvirus 1, Mycoplasma and Chlamydia, and the metabolism of coinfected feline cells
Source: mSystems. 2024 Sep 24;9(10):e00852-24. doi: 10.1128/msystems.00852-24 (PMC11495031; doi:10.1128/msystems.00852-24)
Supplement: Table S1 — Metabolite profile data set. [file msystems.00852-24-s0001.docx]

| **Supplementary data**  **Table S1.** Concentrations of metabolites detected by GC-MS in CRFK cells after inoculation with medium (Mock), single infection with felid herpesvirus 1 (FHV), *M. felis* (M), or *C. felis* (C), or coinfection with *M. felis* and felid herpesvirus 1 (M+FHV), *C. felis* and felid herpesvirus 1 (C+FHV), or *M. felis* and *C. felis* (M+C). | | | | | | | |
| --- | --- | --- | --- | --- | --- | --- | --- |
|  | MOCK | FHV | M | C | M+FHV | C+FHV | M+C |
| Dimethylglycine | -0.42 (0.06) | -0.35 (0.04) | -0.41 (0.05) | -0.38 (0.09) | -0.39 (0.05) | -0.40 (0.05) | -0.39 (0.03) |
| Glyoxylic acid | -0.40 (0.06) | -0.34 (0.05) | -0.40 (0.05) | -0.36 (0.09) | -0.37 (0.04) | -0.39 (0.04) | -0.37 (0.04) |
| Ethanolamine | -0.77 (0.04) | -0.75 (0.11) | -0.67 (0.14) | -0.73 (0.09) | -0.71 (0.08) | -0.81 (0.05) | -0.76 (0.04) |
| Pyruvic acid | -0.06 (0.05) | -0.01 (0.03) | -0.06 (0.05) | -0.18 (0.08) | -0.03 (0.05) | -0.10 (0.04) | -0.08 (0.04) |
| Lactic acid | 1.87 (0.05) | 1.94 (0.08) | 1.87 (0.03) | 1.83 (0.05) | 1.88 (0.04) | 1.82 (0.05) | 1.81 (0.05) |
| Alpha-hydroxyisobutyric acid | -1.00 (0.03) | -0.89 (0.05) | -0.95 (0.05) | -0.85 (0.06) | -0.96 (0.05) | -0.89 (0.11) | -0.92 (0.15) |
| Glycolic acid | 0.08 (0.05) | 0.12 (0.01) | 0.16 (0.05) | 0.11 (0.03) | 0.10 (0.06) | 0.15 (0.05) | 0.07 (0.08) |
| Caproic acid | 0.59 (0.08) | 0.68 (0.04) | 0.62 (0.07) | 0.64 (0.09) | 0.59 (0.01) | 0.69 (0.08) | 0.61 (0.12) |
| Alanine | 1.25 (0.03) | 1.53 (0.06) | 1.37 (0.01) | 1.37 (0.07) | 1.51 (0.05) | 1.48 (0.04) | 1.56 (0.07) |
| Glycine | 1.67 (0.04) | 1.57 (0.07) | 1.64 (0.1) | 1.56 (0.1) | 1.63 (0.07) | 1.52 (0.07) | 1.62 (0.08) |
| Oxalic acid | 0.32 (0.04) | 0.53 (0.06) | 0.63 (0.11) | 0.47 (0.08) | 0.37 (0.15) | 0.42 (0.11) | 0.46 (0.1) |
| Hydroxylamine | 1.93 (0.08) | 2.34 (0.05) | 2.17 (0.12) | 2.16 (0.19) | 2.03 (0.24) | 2.15 (0.14) | 2.21 (0.14) |
| Acetoacetic acid | 0.92 (0.04) | 1.13 (0.03) | 1.06 (0.06) | 1.04 (0.09) | 0.98 (0.16) | 1.02 (0.11) | 1.07 (0.09) |
| Hydroxypropionic acid | 0.09 (0.03) | 0.11 (0.02) | 0.12 (0.05) | 0.11 (0.06) | 0.07 (0.04) | 0.10 (0.02) | 0.05 (0.07) |
| 3-Hydroxybutyric acid | 0.18 (0.08) | 0.18 (0.14) | 0.19 (0.06) | 0.15 (0.12) | 0.19 (0.08) | 0.09 (0.09) | 0.09 (0.02) |
| (S)-3-Hydroxyisobutyric acid | -0.30 (0.07) | -0.28 (0.13) | -0.24 (0.02) | -0.34 (0.06) | -0.29 (0.08) | -0.40 (0.07) | -0.38 (0.02) |
| 3-methyl-2-oxovaleric acid | -1.53 (0.12) | -1.49 (0.29) | -1.25 (0.13) | -1.57 (0.08) | -1.30 (0.09) | -1.51 (0.06) | -1.89 (0.36) |
| Beta-alanine | 0.36 (0.04) | 0.36 (0.06) | 0.34 (0.08) | 0.32 (0.05) | 0.36 (0.05) | 0.34 (0.06) | 0.41 (0.04) |
| 3-hydroxyisovaleric acid | -1.28 (0.19) | -1.30 (0.13) | -1.86 (0.75) | -1.19 (0.21) | -1.45 (0.34) | -1.34 (0.11) | -1.42 (0.29) |
| Ketoleucine | -0.95 (0.08) | -0.98 (0.05) | -0.90 (0.05) | -1.10 (0.06) | -0.84 (0.05) | -1.14 (0.19) | -1.31 (0.08) |
| Valine | 1.73 (0.03) | 1.82 (0.04) | 1.78 (0.03) | 1.78 (0.03) | 1.76 (0.06) | 1.73 (0.03) | 1.84 (0.01) |
| Urea | 0.76 (0.11) | 0.74 (0.14) | 0.74 (0.11) | 0.75 (0.08) | 0.63 (0.07) | 0.68 (0.07) | 0.70 (0.11) |
| Benzoic acid | 1.31 (0.1) | 1.31 (0.04) | 1.24 (0.03) | 1.20 (0.11) | 1.30 (0.12) | 1.34 (0.16) | 1.30 (0.08) |
| Caprylic acid | 0.41 (0.06) | 0.45 (0.05) | 0.32 (0.07) | 0.36 (0.04) | 0.40 (0.08) | 0.44 (0.09) | 0.39 (0.07) |
| Glycerol | 0.53 (0.04) | 0.53 (0.08) | 0.45 (0.07) | 0.49 (0.08) | 0.50 (0.11) | 0.57 (0.17) | 0.43 (0.06) |
| Acetylalanine | 1.89 (0.04) | 1.97 (0.05) | 1.94 (0.03) | 1.96 (0.03) | 1.92 (0.06) | 1.88 (0.02) | 2.01 (0.02) |
| Leucine | 1.91 (0.04) | 1.98 (0.05) | 1.96 (0.03) | 1.97 (0.03) | 1.93 (0.06) | 1.89 (0.02) | 2.02 (0.02) |
| Phosphoric acid | 2.54 (0.05) | 2.72 (0.04) | 2.65 (0.04) | 2.65 (0.01) | 2.66 (0.04) | 2.7 (0.24) | 2.6 (0.11) |
| Isoleucine | 1.76 (0.03) | 1.83 (0.04) | 1.81 (0.03) | 1.82 (0.03) | 1.78 (0.06) | 1.76 (0.03) | 1.87 (0.02) |
| Maleic acid | -1.07 (0.06) | -1.04 (0.04) | -0.96 (0.03) | -0.92 (0.03) | -1.07 (0.1) | -1.07 (0.05) | -0.9 (0.04) |
| Proline | 1.83 (0.03) | 1.90 (0.04) | 1.92 (0.04) | 1.98 (0.02) | 1.82 (0.06) | 1.78 (0.04) | 2.06 (0.02) |
| Phenylacetic acid | -1.48 (0.05) | -1.51 (0.2) | -1.50 (0.07) | -1.63 (0.06) | -1.51 (0.05) | -1.52 (0.11) | -1.45 (0.13) |
| Succinic acid | 0.29 (0.06) | 0.51 (0.12) | 0.44 (0.03) | 0.46 (0.04) | 0.39 (0.06) | 0.42 (0.10) | 0.39 (0.10) |
| Methylsuccinic acid | -1.8 (0.12) | -1.55 (0.07) | -1.74 (0.06) | -1.79 (0.13) | -1.82 (0.19) | -1.72 (0.09) | -1.69 (0.05) |
| Glyceric acid | -0.53 (0.09) | -0.44 (0.06) | -0.35 (0.12) | -0.45 (0.03) | -0.48 (0.09) | -0.50 (0.10) | -0.57 (0.08) |
| Fumaric acid | 0.37 (0.05) | 0.45 (0.04) | 0.47 (0.01) | 0.43 (0.05) | 0.37 (0.07) | 0.27 (0.02) | 0.24 (0.07) |
| Uracil | -0.17 (0.08) | 0.00 (0.16) | -0.35 (0.24) | -0.14 (0.05) | -0.15 (0.05) | -0.06 (0.11) | -0.02 (0.10) |
| Itaconic acid | -1.2 (0.11) | -1.06 (0.06) | -1.12 (0.03) | -1.14 (0.08) | -1.13 (0.05) | -1.19 (0.07) | -1.16 (0.03) |
| Pelargonic acid | 0.73 (0.06) | 0.75 (0.06) | 0.67 (0.04) | 0.69 (0.09) | 0.71 (0.1) | 0.74 (0.09) | 0.69 (0.06) |
| Serine | 2.32 (0.03) | 2.41 (0.04) | 2.36 (0.04) | 2.37 (0.02) | 2.33 (0.06) | 2.27 (0.02) | 2.41 (0.03) |
| Threonine | 2.03 (0.03) | 2.12 (0.04) | 2.07 (0.04) | 2.09 (0.01) | 2.03 (0.06) | 1.98 (0.01) | 2.12 (0.02) |
| O-acetylserine | 0.1 (0.02) | 0.18 (0.04) | 0.14 (0.05) | 0.15 (0.02) | 0.10 (0.05) | 0.05 (0.01) | 0.17 (0.03) |
| Glutaric acid | -1.16 (0.03) | -1.12 (0.06) | -1.07 (0.05) | -1.14 (0.04) | -1.16 (0.08) | -1.15 (0.14) | -1.17 (0.04) |
| Hydroquinone | -1.44 (0.09) | -1.41 (0.06) | -1.61 (0.18) | -1.52 (0.14) | -1.48 (0.09) | -1.46 (0.08) | -1.40 (0.10) |
| Thymine | -1.38 (0.08) | -1.30 (0.12) | -1.61 (0.2) | -1.53 (0.03) | -1.23 (0.32) | -1.41 (0.08) | -1.38 (0.13) |
| Capric acid | -0.2 (0.06) | -0.21 (0.07) | -0.24 (0.01) | -0.25 (0.06) | -0.2 (0.14) | -0.25 (0.06) | -0.28 (0.08) |
| Citramalic acid | -1.31 (0.09) | -1.27 (0.06) | -1.07 (0.27) | -1.22 (0.14) | -1.37 (0.08) | -1.39 (0.06) | -1.42 (0.05) |
| Malic acid | 0.33 (0.04) | 0.39 (0.04) | 0.36 (0.02) | 0.34 (0.05) | 0.32 (0.06) | 0.20 (0.01) | 0.22 (0.04) |
| Niacinamide | 0.48 (0.03) | 0.64 (0.03) | 0.62 (0.08) | 0.57 (0.04) | 0.55 (0.07) | 0.52 (0.05) | 0.63 (0.07) |
| Adipic acid | -0.87 (0.16) | -0.98 (0.13) | -1.07 (0.05) | -1.01 (0.02) | -0.9 (0.22) | -0.97 (0.17) | -1.01 (0.06) |
| Acetylserine | -1.25 (0.00) | -1.13 (0.09) | -1.18 (0.09) | -1.24 (0.15) | -1.11 (0.08) | -1.27 (0.05) | -1.21 (0.08) |
| 3-aminoglutaric acid | 2.46 (0.02) | 2.48 (0.03) | 2.54 (0.05) | 2.52 (0.01) | 2.41 (0.05) | 2.43 (0.03) | 2.59 (0.03) |
| Aspartic acid | 2.44 (0.02) | 2.46 (0.03) | 2.52 (0.05) | 2.50 (0.01) | 2.40 (0.05) | 2.41 (0.02) | 2.58 (0.03) |
| Methionine | 0.21 (0.03) | 0.27 (0.05) | 0.21 (0.05) | 0.22 (0.02) | 0.21 (0.05) | 0.11 (0.02) | 0.23 (0.03) |
| 4-hydroxyproline | 0.64 (0.01) | 0.76 (0.04) | 0.71 (0.05) | 0.71 (0.02) | 0.68 (0.07) | 0.6 (0.01) | 0.72 (0.03) |
| Pyroglutamic acid | 1.76 (0.01) | 1.86 (0.03) | 1.8 (0.06) | 1.82 (0.02) | 1.79 (0.06) | 1.69 (0.02) | 1.84 (0.03) |
| Cytosine | -0.85 (0.04) | -0.72 (0.12) | -1.09 (0.26) | -0.93 (0.2) | -0.84 (0.13) | -0.78 (0.07) | -0.73 (0.17) |
| Gamma-aminobutyric acid | -1.07 (0.03) | -0.65 (0.02) | -0.92 (0.13) | -0.84 (0.12) | -0.9 (0.19) | -0.7 (0.11) | -0.71 (0.11) |
| Cysteine | 0.54 (0.07) | 0.73 (0.05) | 0.71 (0.04) | 0.70 (0.05) | 0.63 (0.14) | 0.67 (0.08) | 0.74 (0.14) |
| 2-Hydroxyglutaric acid | -0.19 (0.01) | -0.16 (0.04) | -0.15 (0.04) | -0.17 (0.01) | -0.17 (0.05) | -0.26 (0.04) | -0.22 (0.04) |
| Creatinine | 0.57 (0.06) | 0.6 (0.06) | 0.71 (0.05) | 0.76 (0.12) | 0.57 (0.12) | 0.69 (0.09) | 0.66 (0.12) |
| Oxoglutaric acid | 0.54 (0.07) | 0.64 (0.06) | 0.6 (0.05) | 0.36 (0.07) | 0.63 (0.08) | 0.43 (0.08) | 0.19 (0.05) |
| O-Phosphoethanolamine | 0.04 (0.05) | -0.10 (0.04) | -0.07 (0.09) | -0.05 (0.1) | -0.16 (0.12) | 0.08 (0.08) | 0.10 (0.02) |
| 3-Hydroxymethylglutaric acid | -1.96 (0.15) | -1.63 (0.07) | -1.85 (0.04) | -1.78 (0.14) | -1.83 (0.23) | -1.7 (0.09) | -1.54 (0.14) |
| Hypotaurine | 1.15 (0.08) | 1.51 (0.04) | 1.41 (0.14) | 1.35 (0.18) | 1.22 (0.21) | 1.30 (0.10) | 1.47 (0.12) |
| Triethanolamine | -1.00 (0.03) | -0.94 (0.19) | -0.52 (0.2) | -0.65 (0.33) | -0.93 (0.13) | -0.95 (0.23) | -0.97 (0.25) |
| Cadaverine | -0.01 (0.10) | 0.36 (0.03) | 0.24 (0.14) | 0.19 (0.20) | 0.06 (0.22) | 0.16 (0.10) | 0.31 (0.12) |
| Glutamic acid | 2.49 (0.02) | 2.60 (0.04) | 2.5 (0.05) | 2.50 (0.02) | 2.52 (0.05) | 2.48 (0.02) | 2.52 (0.02) |
| 5-aminopentanoic acid | -0.61 (0.07) | -0.42 (0.04) | -0.46 (0.1) | -0.39 (0.12) | -0.63 (0.19) | -0.54 (0.09) | -0.38 (0.08) |
| Phenylalanine | 1.32 (0.04) | 1.39 (0.05) | 1.35 (0.05) | 1.37 (0.04) | 1.34 (0.06) | 1.29 (0.02) | 1.41 (0.01) |
| Dodecanoic acid | 0.02 (0.08) | -0.04 (0.04) | -0.06 (0.08) | -0.07 (0.07) | 0.04 (0.14) | -0.16 (0.10) | -0.15 (0.11) |
| Acetylaspartic acid | -0.31 (0.03) | -0.28 (0.04) | -0.18 (0.06) | -0.24 (0.03) | -0.31 (0.05) | -0.28 (0.03) | -0.24 (0.1) |
| Homocysteine | -0.86 (0.02) | -0.98 (0.05) | -0.88 (0.11) | -1 (0.08) | -0.97 (0.07) | -1.12 (0.1) | -1.08 (0.08) |
| Asparagine | -0.49 (0.05) | -0.3 (0.03) | -0.53 (0.08) | -0.34 (0.08) | -0.51 (0.07) | -0.42 (0.09) | -0.23 (0.1) |
| Ribose | -1.23 (0.03) | -1.10 (0.09) | -1.00 (0.06) | -0.95 (0.09) | -1.10 (0.10) | -1.19 (0.04) | -1.11 (0.04) |
| Xylulose | -1.37 (0.07) | -1.23 (0.08) | -1.12 (0.08) | -1.1 (0.16) | -1.11 (0.09) | -1.26 (0.03) | -1.26 (0.09) |
| Cysteine sulfinic acid | -0.94 (0.03) | -0.74 (0.01) | -0.96 (0.13) | -1.1 (0.08) | -0.92 (0.12) | -0.84 (0.07) | -0.79 (0.07) |
| Xylitol | -0.81 (0.11) | -0.83 (0.07) | -0.83 (0.11) | -0.88 (0.09) | -0.73 (0.09) | -1.08 (0.19) | -1.01 (0.14) |
| Levoglucosan | -0.36 (0.1) | -0.34 (0.06) | -0.42 (0.04) | -0.16 (0.08) | -0.15 (0.07) | -0.27 (0.13) | -0.45 (0.09) |
| Aminoadipic acid | -0.6 (0.05) | -0.27 (0.04) | -0.59 (0.01) | -0.46 (0.03) | -0.35 (0.04) | -0.50 (0.05) | -0.45 (0.06) |
| Arabitol | -0.82 (0.08) | -0.78 (0.07) | -0.87 (0.08) | -0.84 (0.07) | -0.67 (0.09) | -0.99 (0.17) | -0.96 (0.16) |
| Rhamnose | -1.33 (0.06) | -1.44 (0.09) | -1.52 (0.1) | -1.41 (0.04) | -1.46 (0.12) | -1.52 (0.11) | -1.39 (0.18) |
| Ribitol | 0.48 (0.08) | 0.37 (0.09) | 0.28 (0.1) | 0.37 (0.12) | 0.41 (0.11) | 0.32 (0.11) | 0.44 (0.07) |
| Cis-aconitate | -0.93 (0.13) | -0.86 (0.14) | -0.84 (0.06) | -1.04 (0.12) | -0.75 (0.04) | -0.85 (0.03) | -0.87 (0.14) |
| 2-Deoxyglucose | 0.11 (0.1) | 0.04 (0.1) | -0.09 (0.09) | 0.01 (0.11) | 0.06 (0.12) | -0.04 (0.11) | 0.08 (0.07) |
| Orotic acid | -0.99 (0.06) | -0.96 (0.1) | -0.91 (0.07) | -0.91 (0.08) | -0.86 (0.18) | -0.94 (0.08) | -1.07 (0.12) |
| Putrescine | -0.86 (0.23) | -0.23 (0.07) | -0.58 (0.17) | -0.40 (0.24) | -0.45 (0.21) | -0.37 (0.23) | -0.49 (0.18) |
| Dihydroxyacetone phosphate | 0.19 (0.04) | 0.14 (0.05) | 0.22 (0.03) | 0.14 (0.07) | 0.14 (0.07) | 0.15 (0.04) | 0.19 (0.05) |
| Glycerol 3-phosphate | 0.07 (0.06) | 0.04 (0.03) | 0.09 (0.04) | 0.04 (0.08) | 0.04 (0.09) | 0.04 (0.05) | 0.09 (0.04) |
| Glutamine | 2.17 (0.09) | 2.22 (0.08) | 2.12 (0.08) | 2.18 (0.04) | 2.14 (0.07) | 2.05 (0.05) | 2.23 (0.04) |
| Azelaic acid | -1.15 (0.13) | -1.14 (0.13) | -1.29 (0.03) | -1.29 (0.07) | -1.12 (0.11) | -1.15 (0.07) | -1.19 (0.04) |
| 3-phosphoglyceric acid | -1.94 (0.25) | -1.82 (0.24) | -1.46 (0.23) | -1.58 (0.21) | -1.15 (0.09) | -1.64 (0.12) | -1.82 (0.24) |
| 2-aminoheptanedioic acid | 0.28 (0.07) | 0.41 (0.05) | 0.36 (0.09) | 0.16 (0.03) | 0.41 (0.05) | 0.26 (0.05) | 0.25 (0.09) |
| Isocitric acid | -0.56 (0.09) | -0.61 (0.15) | -0.53 (0.07) | -0.68 (0.05) | -0.42 (0.08) | -0.59 (0.07) | -0.70 (0.20) |
| Citric acid | 0.85 (0.07) | 0.98 (0.04) | 0.92 (0.08) | 0.73 (0.03) | 0.98 (0.06) | 0.83 (0.04) | 0.82 (0.08) |
| Arginine | -0.25 (0.04) | -0.19 (0.02) | -0.23 (0.06) | -0.33 (0.06) | -0.15 (0.05) | -0.30 (0.04) | -0.20 (0.02) |
| Ornithine | -0.11 (0.08) | 0.08 (0.21) | -0.02 (0.04) | 0.02 (0.02) | -0.18 (0.07) | -0.03 (0.12) | 0.08 (0.10) |
| Glycylglycine | -0.90 (0.05) | -0.76 (0.08) | -0.79 (0.05) | -0.78 (0.09) | -0.82 (0.14) | -0.89 (0.06) | -0.83 (0.13) |
| Myristic acid | 0.75 (0.06) | 0.72 (0.01) | 0.69 (0.08) | 0.69 (0.05) | 0.72 (0.05) | 0.69 (0.06) | 0.67 (0.02) |
| Fructose | -0.59 (0.15) | -0.89 (0.17) | -0.71 (0.12) | -0.70 (0.21) | -0.75 (0.1) | -0.76 (0.24) | -0.66 (0.16) |
| Sorbose | -0.81 (0.16) | -1.42 (0.31) | -0.96 (0.15) | -0.94 (0.12) | -1.07 (0.07) | -0.98 (0.21) | -0.86 (0.13) |
| Pyridoxal | -1.04 (0.13) | -1.01 (0.13) | -0.92 (0.08) | -1.01 (0.12) | -0.93 (0.09) | -1.08 (0.05) | -1.01 (0.06) |
| Galactose | -0.42 (0.13) | -0.63 (0.14) | -0.55 (0.07) | -0.56 (0.09) | -0.59 (0.1) | -0.61 (0.13) | -0.45 (0.17) |
| Glucose | -0.61 (0.14) | -0.75 (0.03) | -0.82 (0.18) | -0.73 (0.06) | -0.72 (0.13) | -0.76 (0.14) | -0.57 (0.17) |
| Ureidosuccinic acid | -1.13 (0.14) | -0.99 (0.07) | -1.16 (0.25) | -1.33 (0.15) | -1.10 (0.16) | -1.06 (0.02) | -1.24 (0.13) |
| Lysine | 0.28 (0.08) | 0.63 (0.01) | 0.39 (0.11) | 0.47 (0.13) | 0.42 (0.14) | 0.41 (0.08) | 0.55 (0.08) |
| Tyramine | 0.28 (0.08) | 0.62 (0.02) | 0.40 (0.10) | 0.45 (0.14) | 0.39 (0.13) | 0.40 (0.08) | 0.53 (0.08) |
| 1-Hexadecanol | -0.40 (0.12) | -0.40 (0.12) | -0.50 (0.1) | -0.47 (0.13) | -0.41 (0.1) | -0.35 (0.07) | -0.41 (0.1) |
| Tyrosine | 0.9 (0.04) | 0.94 (0.05) | 0.85 (0.07) | 0.90 (0.03) | 0.90 (0.06) | 0.83 (0.03) | 0.96 (0.00) |
| Mannitol | -0.02 (0.11) | -0.07 (0.06) | -0.03 (0.1) | 0.00 (0.06) | 0.03 (0.13) | -0.13 (0.15) | -0.09 (0.12) |
| Sorbitol | -0.17 (0.13) | -0.24 (0.07) | -0.22 (0.09) | -0.15 (0.08) | -0.13 (0.11) | -0.29 (0.14) | -0.28 (0.14) |
| Galactitol | -0.02 (0.13) | -0.05 (0.07) | -0.03 (0.09) | 0.02 (0.07) | 0.02 (0.15) | -0.09 (0.13) | -0.07 (0.12) |
| Pantothenic acid | -0.02 (0.03) | 0.07 (0.05) | 0.02 (0.05) | 0.13 (0.02) | 0.07 (0.04) | 0.12 (0.03) | 0.12 (0.03) |
| Scyllo-inositol | -0.26 (0.24) | -0.25 (0.28) | -0.11 (0.14) | -0.23 (0.18) | -0.30 (0.20) | -0.29 (0.25) | -0.27 (0.19) |
| Palmitoleic acid | 0.29 (0.02) | 0.31 (0.05) | 0.27 (0.09) | 0.24 (0.02) | 0.30 (0.04) | 0.24 (0.08) | 0.27 (0.02) |
| Palmitic acid | 1.85 (0.13) | 1.78 (0.02) | 1.75 (0.07) | 1.77 (0.09) | 1.72 (0.07) | 1.75 (0.07) | 1.77 (0.04) |
| Ribose 5-phosphate | -0.29 (0.05) | -0.24 (0.05) | -0.09 (0.05) | -0.16 (0.08) | -0.22 (0.1) | -0.33 (0.04) | -0.25 (0.07) |
| Myo-inositol | 2.81 (0.01) | 2.78 (0.04) | 2.91 (0.04) | 2.84 (0.01) | 2.77 (0.06) | 2.73 (0.02) | 2.83 (0.04) |
| Acetylgalactosamine | 2.01 (0.01) | 1.98 (0.04) | 2.11 (0.03) | 2.05 (0.01) | 1.97 (0.06) | 1.93 (0.02) | 2.04 (0.04) |
| Acetylmannosamine | 1.58 (0.01) | 1.56 (0.04) | 1.68 (0.04) | 1.62 (0.01) | 1.55 (0.06) | 1.51 (0.02) | 1.61 (0.04) |
| Heptadecanoic acid | -0.01 (0.11) | -0.02 (0.04) | -0.06 (0.08) | -0.09 (0.09) | -0.04 (0.04) | -0.01 (0.04) | -0.03 (0.09) |
| Octadecanol | 0.21 (0.23) | 0.11 (0.12) | 0.18 (0.21) | 0.16 (0.18) | 0.18 (0.13) | 0.16 (0.19) | 0.18 (0.22) |
| Norepinephrine | -0.53 (0.06) | -0.27 (0.01) | -0.40 (0.12) | -0.46 (0.14) | -0.50 (0.20) | -0.51 (0.06) | -0.40 (0.12) |
| Linoleic acid | 0.62 (0.13) | 0.27 (0.15) | 0.34 (0.26) | 0.30 (0.37) | 0.50 (0.19) | 0.23 (0.25) | 0.20 (0.21) |
| Oleic acid | 1.27 (0.06) | 1.26 (0.03) | 1.20 (0.08) | 1.18 (0.05) | 1.24 (0.05) | 1.18 (0.08) | 1.19 (0.04) |
| Elaidic acid | 0.37 (0.03) | 0.38 (0.05) | 0.26 (0.11) | 0.33 (0.04) | 0.35 (0.05) | 0.33 (0.09) | 0.34 (0.01) |
| Cystathionine | -1.25 (0.07) | -1.38 (0.26) | -1.60 (0.26) | -1.45 (0.2) | -1.37 (0.16) | -1.5 (0.15) | -1.22 (0.09) |
| Stearic acid | 1.84 (0.12) | 1.78 (0.02) | 1.72 (0.06) | 1.75 (0.08) | 1.73 (0.06) | 1.75 (0.06) | 1.78 (0.03) |
| Tryptophan | -0.53 (0.08) | -0.62 (0.08) | -0.73 (0.2) | -0.65 (0.13) | -0.56 (0.18) | -0.64 (0.08) | -0.5 (0.02) |
| Tryptamine | -0.88 (0.06) | -0.61 (0.04) | -0.71 (0.07) | -0.81 (0.13) | -0.78 (0.14) | -0.84 (0.11) | -0.68 (0.13) |
| Glucose 6-phosphate | -1.41 (0.41) | -2.16 (0.51) | -0.86 (0.22) | -0.68 (0.19) | -1.03 (0.12) | -1.66 (0.14) | -1.99 (0.28) |
| Arachidonic acid | 0.53 (0.02) | 0.57 (0.02) | 0.53 (0.07) | 0.50 (0.01) | 0.54 (0.06) | 0.50 (0.06) | 0.50 (0.04) |
| Eicosapentaenoic acid | -0.34 (0.06) | -0.33 (0.06) | -0.34 (0.16) | -0.47 (0.17) | -0.28 (0.04) | -0.45 (0.1) | -0.32 (0.05) |
| Inositol phosphate | 0.59 (0.07) | 0.65 (0.15) | 0.42 (0.15) | 0.5 (0.14) | 0.59 (0.05) | 0.64 (0.07) | 0.64 (0.08) |
| Glycerol 1-octadecanoate | 0.57 (0.02) | 0.29 (0.29) | 0.64 (0.04) | 0.58 (0.02) | 0.05 (0.62) | 0.49 (0.04) | 0.41 (0.30) |
| Sucrose | 0.75 (0.38) | 0.58 (0.42) | 0.53 (0.55) | 1.50 (0.08) | 0.17 (0.15) | 1.50 (0.1) | 1.65 (0.37) |
| Xanthylic acid | 0.00 (0.13) | -0.10 (0.13) | 0.27 (0.05) | 0.22 (0.07) | 0.18 (0.13) | -0.07 (0.08) | -0.09 (0.17) |
| Cholesterol | 1.12 (0.02) | 1.21 (0.04) | 1.19 (0.04) | 1.14 (0.03) | 1.15 (0.05) | 1.12 (0.03) | 1.12 (0.03) |
| Lathosterol | -0.79 (0.01) | -0.75 (0.04) | -0.69 (0.06) | -0.81 (0.03) | -0.79 (0.05) | -0.85 (0.03) | -0.80 (0.06) |
| Dimethylglycine | -0.42 (0.06) | -0.35 (0.04) | -0.41 (0.05) | -0.38 (0.09) | -0.39 (0.05) | -0.40 (0.05) | -0.39 (0.03) |

Median normalised and log transformed data presented as means (standard deviation)
